# TRAP1 Regulation of Cancer Metabolism: Dual Role as Oncogene or Tumor Suppressor

**DOI:** 10.3390/genes9040195

**Published:** 2018-04-05

**Authors:** Danilo Swann Matassa, Ilenia Agliarulo, Rosario Avolio, Matteo Landriscina, Franca Esposito

**Affiliations:** 1 Department of Molecular Medicine and Medical Biotechnology, University of Naples Federico II, 80131 Naples, Italy; daniloswann.matassa@unina.it; 2 Institute of Protein Biochemistry (IBP), National Research Council, 80131 Naples, Italy; ilenia.agliarulo@gmail.com; 3 Gene Regulation, Stem Cells and Cancer Programme, Centre for Genomic Regulation (CRG), 08003 Barcelona, Spain; rosario.avolio@crg.eu; 4 Medical Oncology Unit, Department of Medical and Surgical Sciences, University of Foggia, 7100 Foggia, Italy; 5 Laboratory of Pre-clinical and Translational Research, IRCCS, Referral Cancer Center of Basilicata, 85028 Rionero in Vulture, Italy

**Keywords:** TRAP1, tumor cell metabolism, oxidative phosphorylation

## Abstract

Metabolic reprogramming is an important issue in tumor biology. An unexpected inter- and intra-tumor metabolic heterogeneity has been strictly correlated to tumor outcome. Tumor Necrosis Factor Receptor-Associated Protein 1 (TRAP1) is a molecular chaperone involved in the regulation of energetic metabolism in cancer cells. This protein is highly expressed in several cancers, such as glioblastoma, colon, breast, prostate and lung cancers and is often associated with drug resistance. However, TRAP1 is also downregulated in specific tumors, such as ovarian, bladder and renal cancers, where its lower expression is correlated with the worst prognoses and chemoresistance. TRAP1 is the only mitochondrial member of the Heat Shock Protein 90 (HSP90) family that directly interacts with respiratory complexes, contributing to their stability and activity but it is still unclear if such interactions lead to reduced or increased respiratory capacity. The role of TRAP1 is to enhance or suppress oxidative phosphorylation; the effects of such regulation on tumor development and progression are controversial. These observations encourage the study of the mechanisms responsible for the dualist role of TRAP1 as an oncogene or oncosuppressor in specific tumor types. In this review, TRAP1 puzzling functions were recapitulated with a special focus on the correlation between metabolic reprogramming and tumor outcome. We wanted to investigate whether metabolism-targeting drugs can efficiently interfere with tumor progression and whether they might be combined with chemotherapeutics or molecular-targeted agents to counteract drug resistance and reduce therapeutic failure.

## 1. Introduction

Although cancer has historically been considered a proliferation-related disorder, emerging evidence suggests that it could also arise from metabolic dysfunction [1]. Reprogramming of energy metabolism is a biological feature acquired during the multistep process of tumor development. Growing tumors must satisfy the bioenergetic and biosynthetic demands of increased cell proliferation and survive the environmental fluctuations in nutrient and oxygen availability when tumor growth exceeds the delivery capabilities of the existing blood vessels [2]. This metabolic rewiring is crucial in cancer transformation and progression. Mutated oncogenes and tumor suppressor genes are responsible for alterations in metabolic signaling pathways: several oncogenes, such as MYC Proto-Oncogene, Hypoxia Inducible Factor 1 (HIF1), Phosphatidylinositol-4,5-Bisphosphate 3-Kinase (PI3K), AKT Serine/Threonine Kinase (AKT) and Mechanistic Target Of Rapamycin Kinase (mTOR), can stimulate transcription of genes encoding for proteins in the glycolysis and glutaminolysis pathways and induce the loss of p53 functions [3]. However, the preference of rapidly proliferating cells for aerobic glycolysis rather than oxidative phosphorylation, the so-called Warburg effect [4], is not a universal feature of cancer anymore. Increasing evidence has shown that some tumors rely instead on oxidative metabolism to meet their increased bioenergetic demands [5] and that different metabolic profiles are defined by histotypes [6] and tumor progression. In support, (1) THP-1 monocytic cells, resistant to 2-Deoxy-d-glucose and sensitive to oligomycin, were reported as an oxidative leukemia cell line [7]; (2) the most aggressive ovarian cancer cell lines rely on glutamine rather than glucose [8] and (3) respiratory function has been reported as being essential for tumorigenic and metastatic potential of breast cancer and melanoma cells [9]. Furthermore, an analysis aimed at investigating the dominant metabolic type in triple negative breast cancer, which is associated with aggressive tumor behavior and poor outcomes, revealed that glycolysis and mitochondrial metabolism-related proteins are both expressed and that the predominant glycolytic or non-glycolytic phenotype varies according to the type of triple negative breast cancer analyzed [10]. This complex scenario suggests that genes involved in pathways controlling energetic metabolism could have context-dependent roles, according to the metabolic profile of each specific tumor type.

In this review, we discuss the role of the molecular chaperone Tumor Necrosis Factor (TNF) Receptor-associated Protein 1 (TRAP1), whose functions in the regulation of energetic metabolism in cancer cells have been highlighted by several research groups, focusing on its oncogenic or oncosuppressive potential role, according to the metabolic need of specific tumor types.

## 2. TRAP1 Background

TRAP1 is a member of the Heat Shock Protein 90 (HSP90) family, the only one showing a predominant but not exclusive mitochondrial localization, having different roles in cancer, neurodegeneration and other apparently unrelated diseases [11]. TRAP1 was firstly described as a TNF-Receptor Associated Protein [12], as a chaperone for the retinoblastoma protein during mitosis and after heat shock [13] and as a factor stabilizing Cyclophilin 40 (CypD) to prevent permeability transition pore opening and thus apoptosis [14]. However, TRAP1 has emerged as both a critical regulator of mitochondrial respiration, through the direct binding to respiratory complexes [15,16,17] and a regulator of cytoplasmic protein synthesis through its binding to both ribosomes and translation factors [18]. In particular, the cytoplasmic pool of TRAP1 supports a switch from a preferential, canonical cap-dependent translation to enhanced Internal Ribosome Entry Site-mediated translation [19]. These regulations lead to the modulation of the expression of mitochondrial proteins involved in energy metabolism (ATP synthase, β subunit) and calcium balance (Sorcin). This suggests a link between the different TRAP1 functions, as suggested by its alternative and multiple locations [20]. Overall, TRAP1: (1) contributes to the regulation of energy metabolism by directly binding to complexes II and IV of the mitochondrial respiratory chain [21]; (2) is part of a pro-survival signaling pathway aimed at protecting mitochondria against the toxic effects of oxidants and anticancer drugs [22]; and (3) participates in protein homeostasis control by directly regulating translation and co-translational protein degradation, binding either component of the translational machinery and the proteasome [11]. However, whether TRAP1 roles are oncogenic or not is still a matter for debate. TRAP1 levels are elevated in several malignancies, including glioblastoma, colon, breast, prostate and lung tumors and has been correlated with drug resistance [23]. Conversely, it is downregulated in specific tumors, some of which with predominant oxidative metabolism, such as ovarian, bladder, cervical and renal cancers [16,24]. The role of TRAP1 in metabolic reprogramming is also controversial: its ability to enhance [15,16] or suppress [17] the switch from oxidative phosphorylation to aerobic glycolysis, known as the Warburg effect, seems to be context-dependent and different in tumors with different histotypes. This is particularly relevant since, contrary to conventional wisdom, functional mitochondria are essential for cancer cells. Accordingly, current knowledge on the biology of TRAP1 suggests that it favors the oncogenic phenotype in glycolytic tumors [25] but is negatively selected in tumors preferentially relying on oxidative metabolism [26].

## 3. TRAP1 Regulation of Energetic Metabolism

The first evidence supporting the TRAP1 control of mitochondrial metabolism was reported by Sciacovelli et al. [15], who demonstrated that tumor cells expressing high levels of TRAP1 had a predominant Warburg phenotype. The results showed that TRAP1 is bound to complex II and IV of the electron transport chain and inhibits the activity of Succinate dehydrogenase (SDH) in Saos-2 osteosarcoma cells but it does not affect the activity of cytochrome oxidase nor the protein levels of complex II and IV and mitochondrial mass. In support of these data, TRAP1 expression was inversely correlated to SDH activity in colon cancer specimens, in which TRAP1 expression was higher in comparison with the relative healthy surrounding mucosas. Moreover, TRAP1 knockdown significantly increased the oxygen consumption rate (OCR) in Saos-2 cells, whereas glycolysis was the main adenosine triphosphate (ATP) source in control cells. In the reverse approach, overexpression of TRAP1 in non-transformed fibroblasts reduced mitochondrial respiration and mimicked the respiratory pattern of cancer cells. Considering that succinate accumulation favors HIF1 alpha subunit (HIF1α) stabilization by inhibiting the prolyl-hydroxylases, which are responsible for HIF1α ubiquitin-dependent degradation, HIF1α expression levels were analyzed in focus forming assays. Results showed that succinate accumulated in TRAP1-expressing cells, as opposed to TRAP1-knockdown cells and that HIF1α was only detectable in the first cell population. Accordingly, tumor cells xenografted with TRAP1-expressing Saos-2 cells had detectable levels of HIF1α, whereas HIF1α downmodulation prevented focus-forming in TRAP1-expressing tumor cells and in mouse embryonic fibroblasts (MEFs) transfected with a TRAP1-expressing construct. In conclusion, this work suggests that TRAP1 is per se able to induce neoplastic growth by stabilizing HIF1α via a metabolic mechanism involving accumulation of succinate [15].

Mechanistically, Yoshida et al [16] showed that TRAP1 reduces mitochondrial respiration and ATP production. TRAP1 knockout (KO) in mouse adult fibroblasts (MAFs) increased both basal OCR and maximum respiratory capacity, also decreasing glycolysis. Re-introduction of TRAP1 in KO MAFs restored basal OCR levels, confirming that TRAP1 KO cells mostly use oxidative phosphorylation rather than glycolysis for ATP production. Consequently, levels of glycolytic metabolites were lower in TRAP1 KO cells, whereas levels of TCA cycle intermediates and anaplerotic substrates, fatty acid oxidation and the nicotinamide adenine dinucleotide (NAD^+^/NADH) ratio were higher. These data strongly suggest that cells not expressing TRAP1 activate the tricarboxylic acid (TCA) cycle independently from glucose metabolism. Accordingly, these cells also show higher complex IV activity and global ATP levels. Analogous results were obtained in HeLa and HCT116 tumor cell lines [16]. In agreement with previous reports, the reduced mitochondrial respiration shown by TRAP1-expressing cells contributed to reduce the reactive oxygen species (ROS) levels in both MAFs and HCT116, thus proving that TRAP1 KO cells are constitutively exposed to elevated oxidative stress. Finally, these authors demonstrated that TRAP1 at least partially regulates mitochondrial respiration, through directly regulating mitochondrial SRC Proto-Oncogene (c-Src), which leads to increased Tyr-416 phosphorylation of c-Src upon TRAP1 suppression. Accordingly, TRAP1 preferentially binds the inactive form of c-Src, suggesting that TRAP1 inhibits c-Src function in mitochondria [16]. Consistent with these data, c-Src overexpression significantly increases mitochondrial OCR but simultaneous overexpression of TRAP1 reduces this effect.

**Figure 1:**
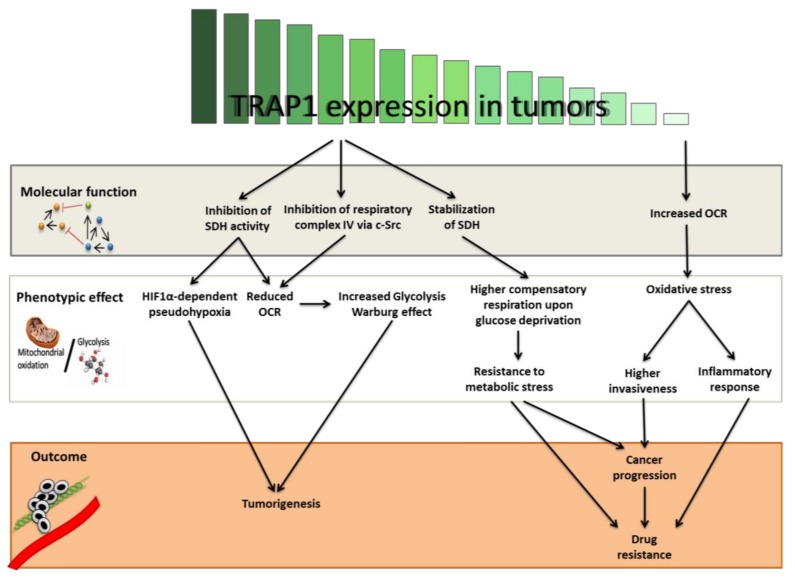
Hierarchical effects of Tumor Necrosis Factor Receptor-associated Protein 1 (TRAP1) expression levels on tumor development and progression through the regulation of energetic metabolism. TRAP1 binds respiratory complexes in the mitochondrial matrix and regulates their activity, either directly or indirectly via c-Src (grey box). This regulation alternatively leads to (i) a switch toward a more glycolytic (Warburg) phenotype, (ii) an enhanced ability to handle metabolic stress through the use of spare respiratory capacity and (iii) increased oxidative stress (white box). As a result, tumors with high glycolytic metabolism or limiting nutrient availability may gain the advantage of a high TRAP1 expression. Conversely, tumors mostly relying on oxidative metabolism may counter-select TRAP1 expression for progression and development of resistance and metastases (orange box). OCR: oxygen consumption rate; SDH: Succinate dehydrogenase; c-Src: SRC Proto-Oncogene; HIF1α: Hypoxia Inducible Factor 1 Alpha Subunit.

Chae et al. [17] demonstrated that TRAP1 interacts with Succinate dehydrogenase B (SDHB) in tumor mitochondria. This interaction prevents the stress-induced SDHB loss of stability. Starting with this result, the authors then asked whether TRAP1 is important for the mitochondrial respiration of prostate cancer cells and found that exposure to the small molecule Gamitrinib, which inhibits both mitochondrial HSP90 and TRAP1, led to decreased OCR and that TRAP1 silencing halted the increase of compensatory OCR induced by glucose deprivation. This mitochondrial network provides high TRAP1-expressing tumor cells the ability to use spare respiratory capacity given limited glucose availability, providing stronger ability to handle metabolic stress. This seems to have important consequences for tumor progression and the development of metastases (Figure 1). The mitochondrial HSP90/TRAP1 network enhances tumor cell invasion during low nutrient and metastatic dissemination [27,28].

Although other studies further investigated the correlation between TRAP1 and energetic metabolism, if TRAP1 enhances or suppresses mitochondrial respiration under different conditions is still unclear. Matassa et al. [26] demonstrated that TRAP1 silencing in ovarian cancer cells increases OCR, without affecting glycolysis. Consequently, matched isogenic cisplatin sensitive and resistant cell lines, derived from the same patient before and after chemotherapy, showed lower levels of TRAP1, increased OCR and decreased glycolysis in the drug resistant cells compared with the drug-sensitive counterpart [26]. A causal relationship between low TRAP1 and increased OCR was observed, since TRAP1 silencing in both sensitive and resistant cells increased mitochondrial respiration [26]. Conversely, in two different breast cancer cell lines, stable knockdown of TRAP1 via short hairpin RNA (shRNA) was responsible for a decrease in OCR [29].

The evidence that the correlation between TRAP1 expression and mitochondrial metabolism is complex arises from two observations: (1) in a non-cancerous system of mouse cardiomyocites, TRAP1 overexpression did not change the activities of complexes I–IV but the activities of complexes III and IV were preserved, while adversely affecting complex II activity in the same cells after simulated ischemia/reperfusion injury [30] and (2) TRAP1 −/− nontransformed MEFs exhibited no changes in complexes I or II but increased the activity of complexes III and IV, resulting in increased OCR, while TRAP1 −/− hepatocytes or MEFs dramatically switched their metabolism to aerobic glycolysis compared with WT controls [31]. However, this confusing evidence was challenged by the same authors, who observed that shRNA-mediate knockdown of TRAP1 in HeLa cells initiated metabolic reprogramming, with increased OCR and higher ATP production compared with shRNA controls.

## 4. TRAP1 as an Oncogene

The first formal demonstration of an asymmetrical distribution of TRAP1 in normal versus malignant tissues was provided by a comparative immunohistochemistry analysis of primary mouse tumor specimens and their matched normal tissues in vivo, showing that TRAP1 expression was higher in tumor cells compared to the normal matched epithelia [14]. These observations were strongly supported by evidence that TRAP1 regulates mitochondrial permeability transition in response to apoptotic stimuli through the interaction with CyclophilinD, an immunophilin that mediates mitochondrial cell death [14]. TRAP1 is mainly located inside the mitochondria of cancerous tissues, where the cytosolic HSP90 is also actively translocated via an unknown mechanism, forming a complex with CyclophilinD, inhibiting its function. Disruption of this complex by HSP90 ATPase antagonists directed to the mitochondria caused sudden collapse of mitochondrial function and apoptosis [14].

Proceeding these observations, TRAP1 was found to be dysregulated in several human cancers. In this study, we briefly summarize the best-characterized contributions of TRAP1 in glioblastoma, prostate, colorectal, breast and non-small cell lung cancers (Table 1).

One of the first studies on TRAP1 as a tumor biomarker was completed in glioblastoma, one of the most aggressive invasive brain tumors, highly resistant to therapy and heterogeneous at both the molecular and histological levels. Immunohistochemical analysis of human specimens from grade IV revealed that TRAP1 was highly expressed in the tumor cell population. Adjacent normal astrocytes did not contain TRAP1, whereas a low level of TRAP1 expression was detected in neurons. Treatment of glioblastoma cells with Shepherdin, a mitochondrial-permeable inhibitor of HSP90 ATPase activity, triggered glioblastoma cell death and suppressed intracranial glioma growth in mouse models [36].

Some studies focused on prostate cancer. TRAP1, along with mitochondria-localized HSP90, were shown to be abundantly and ubiquitously expressed in both localized and metastatic prostate cancers but were largely undetectable in normal prostate or benign prostatic hyperplasia in vivo. TRAP1 silencing in androgen-independent prostate cancer cells enhanced apoptosis and treatment with mitochondria-directed HSP90 inhibitors (Gamitrinibs) selectively caused the death of prostate cancer cells [40]. Transgenic mice expressing TRAP1 in the prostate developed epithelial hyperplasia and cellular atypia and, when examined in a Phosphatase And Tensin Homolog (Pten)+/− background, a common alteration in human prostate cancer, showed accelerated incidence of invasive prostatic adenocarcinoma, whereas deletion of TRAP1 delayed prostatic tumorigenesis. Accordingly, global profiling of Pten+/− TRAP1 transgenic mice by RNA sequencing and reverse phase protein array modulated oncogenic networks of cell proliferation, apoptosis, cell motility and DNA damage [41], further supporting the role of TRAP1 as a driver of prostate cancer, with potential for novel therapeutic approaches.

However, prostate cancer was not the only cancer type investigated for the role of TRAP1 in cancer progression. Early evaluation of TRAP1 expression in a limited number of human colorectal carcinomas showed up-regulation compared with normal matched peritumoral mucosa in 65% of cases [33]. Accordingly, TRAP1 levels increased in colorectal carcinoma cells resistant to 5-fluorouracil, oxaliplatin and irinotecan and, in turn, its overexpression led to drug-resistance. Accordingly, immunohistochemistry analyses of 714 cases of colorectal carcinoma have shown that high TRAP1 expression was observed in 79% of cases [34]. TRAP1 expression significantly increased in colorectal cancer at the advanced pathological stage, being significantly correlated with poor survival rates, although only marginally associated with lymph node involvement and tumor differentiation. Accordingly, a protein signature predictive of poor prognoses in advanced disease was identified by proteomic analysis of the TRAP1 protein network in human colorectal carcinomas. This approach strongly correlated the upregulation of TRAP1 and six of its interactors with poor outcomes in metastatic cancer [35]. In addition, TRAP1 was upregulated early in colorectal carcinogenesis, at the transition between low- and high-grade adenomas [35].

Mechanistically, TRAP1 has been shown to regulate two signaling pathways responsible for colorectal carcinogenesis at the molecular level, such as B-Raf Proto-Oncogene (BRAF) and Wnt/βCatenin signaling. Indeed, TRAP1 modulates the expression of the BRAF oncogene and the two proteins interact in colorectal cancer cells, being frequently co-expressed in human colorectal carcinomas [42]. Reciprocally, BRAF cytoprotective signaling involves the TRAP1-dependent inhibition of the mitochondrial apoptotic pathway. TRAP1 targeting by the mitochondria-directed HSP90 inhibitor Gamitrinib induced apoptosis and inhibited colony formation in BRAF-driven colorectal carcinoma cells, which are known to be poorly responsive to anticancer therapies [43]. Our group was the first to suggest a role for TRAP1 in stemness. TRAP1 expression is enhanced in stem cells located at the bottom of intestinal crypts and in cancer stem cells separated from colorectal cancer cell lines. Moreover, TRAP1 silencing in HCT116 colorectal cancer cells resulted in the loss of the stem-like signature [44]. Notably, TRAP1 regulated the phosphorylation and degradation of βCatenin, thus positively controlling the Wnt/βCatenin pathway, which is activated in colorectal cancers with high TRAP1 expression [44].

Analogously, TRAP1 has been found to be aberrantly upregulated in breast tumors compared with control tissues. Its expression in human breast cancer specimens was inversely correlated with tumor grade. TRAP1 knockdown in breast cancer cells also sensitized cells to lethal stimuli and inhibited tumor growth [29]. Moreover, TRAP1 upregulation in breast cancer cells led to paclitaxel resistance, a drug commonly used in breast cancer treatment that alters microtubule assembly and induces endoplasmic reticulum (ER) stress [32] and to genotoxic agents, such as anthracyclins [45]. TRAP1 expression level was higher in paclitaxel- and anthracyclin-resistant cell lines compared with non-resistant counterparts but its knockdown or inhibition by Gamitrinib restored drug sensitivity [32].

Finally, TRAP1 expression was investigated in lung cancer. TRAP1 knockdown reduced cell growth and clonogenic cell survival and impaired mitochondrial function in non-small cell lung cancer cells. A moderate TRAP1 staining by immunohistochemical analysis was found in normal bronchial mucosa, as opposed to the adjacent tumor. High TRAP1 expression was associated with an increased risk of disease recurrence [37].

Aligned with the evidence above, the expression of key cell cycle regulators, like Cyclin Dependent Kinase 1 (CDK1) and Mitotic Arrest Deficient 2 (MAD2), was modulated by TRAP1 in a large cohort of colorectal, breast and lung adenocarcinoma cell lines, with consequent effects on mitotic entry [46]. TRAP1 expression was significantly correlated with CDK1 and MAD2 expression and the CDK1 inhibitor RO-3306 is less active in a TRAP1-high background. This suggests that, at least in these tumors, the TRAP1-dependent regulation of cell proliferation relies on metabolic control, in keeping with previous observations in breast and lung cancer cell lines [47].

Relevant in vitro studies formally demonstrated molecular mechanisms and pathways involved in TRAP1 control of cancer progression. Sciacovelli et al. [15] suggested that TRAP1 promotes neoplastic transformation in different cell systems, showing that different cancer cell lines lose their transforming potential during in vitro focus forming assays, soft agar assays and after injection in nude mice upon TRAP1 silencing, whereas TRAP1 overexpression conferred transforming potential to fibroblasts. This TRAP1 transforming potential is strictly dependent on its mitochondrial localization and organelle-correlated functions, since the overexpression of a TRAP1 deletion mutant lacking the mitochondrial targeting sequence does not reproduce the abovementioned phenotype.

Consistent with these findings, we previously demonstrated a mechanistic link between high TRAP1 expression, activation of Extracellular Signal-Regulated Kinases (ERK) signaling and cell cycle progression [42]. Another study demonstrated a mechanistic connection between deregulated Ras/ERK signaling and the pro-neoplastic metabolic switch from oxidative phosphorylation to glycolysis, orchestrated by TRAP1 through the inhibition of SDH [48]. This study showed that active ERK1/2 interacts with TRAP1 and SDH in the mitochondria of neurofibromin-deficient cells, where ERK-dependent phosphorylation of TRAP1 enhances TRAP1-SDH binding and inhibition of SDH and, consequently, tumorigenic cell potential.

## 5. TRAP1 as a Tumor Suppressor

Compelling evidence suggests a link between TRAP1 antioxidant function [49] and its tumor suppressing activity. However, the correlations between TRAP1 molecular functions and their effect in tumor cells are still controversial; some authors suggest that TRAP1 oncogenic potential also depends on the chaperone scavenging activity [50] and not only on its ability to induce pseudohypoxia through the inhibition of SDH, with subsequent accumulation of the HIF1α-stabilizing metabolite succinate. Conversely, considering that elevated ROS can also favor cell invasion [51], Yoshida et al. [16] hypothesized that TRAP1 silencing in cancer cells could enhance migration, contributing to a more severe malignant phenotype. Accordingly, these authors demonstrated that cell invasiveness increases in mouse fibroblasts and in several human cell lines upon TRAP1 knockdown or knockout. Since the increased invasiveness is affected by ROS scavenging drugs, the authors proposed the existence of a direct link between low TRAP1 expression, elevated ROS levels and activation of cell migration and invasion. The impact of reduced TRAP1 expression on in vitro cell invasion led to the hypothesis that some more aggressive, metastatic, or late-stage tumors may have lower TRAP1 levels than less advanced tumors. In agreement with this hypothesis, the authors reported an inverse correlation between TRAP1 expression and tumor stage and cervical, bladder and clear cell renal cell carcinomas [16]. Cervical carcinoma has been described as a type of cancer that mainly relies on oxidative phosphorylation for its energy production [52]. Therefore, these observations suggest that the role of TRAP1 in cancer progression depends on the tumor features and that TRAP1 inhibition alone could not be considered a viable treatment strategy for cancers that rely on oxidative phosphorylation [16].

The hypothesis that TRAP1 may act as a tumor suppressor depending on the tumor type and its relative context was initially suggested by a study of 208 patients affected by ovarian cancer. The authors showed that high TRAP1 immunohistochemical staining was positively correlated with chemotherapy response and with overall survival [24]. Moreover, they found a significant correlation between high TRAP1 expression and estrogen receptor α levels. This last observation was also supported by the previous demonstration of increased TRAP1 expression in Letrozole-responsive cancers compared to Letrozole-insensitive cancers [38]. This evidence, together with the previous inclusion of TRAP1 in a list of estrogen-regulated genes [53], prompted us to focus on the correlation between TRAP1 and tumor outcome in gynecological malignancies. We showed that TRAP1 expression is inversely correlated with tumor stage and grade and is directly associated to better survival in a large datasets of ovarian cancer patients [26]. In this tumor, the *TRAP1* gene is deleted in the late stages of the disease, suggesting genetic loss as the most likely mechanism for the decrease in TRAP1 expression as ovarian cancer progresses [39]. This mechanism could either be causally related to the metabolic remodeling of the tumor or be part of the selection process driven by survival advantage. In keeping with patient data, platinum resistant cell lines show lower TRAP1 levels than their sensitive counterparts in cellular models of high grade serous ovarian cancer isogenic matched paired cell lines derived from the same patient before and after platinum-based therapy. Accordingly, the metabolic reprogramming of oxidative phosphorylation initiates an inflammatory status responsible for drug resistance in these cells [26]. Moreover, in high-grade serous ovarian cancers, TRAP1 expression is mainly found in peritoneal biopsies distant from the primary tumors [39], suggesting that low TRAP1 expressing cancers may be more prone to spread from the primary site. TRAP1 expression was also inversely correlated to the expression of several markers of epithelial-mesenchymal transition in both ovarian cancer cells and tissues [39].

This puzzling function of TRAP1 in ovarian cancer fits well with the demonstration of TRAP1 being responsible for, or involved in, the shift of glycolytic metabolism toward oxidative phosphorylation [26]. Indeed, as a consequence of this metabolic remodeling, platinum resistance in ovarian cancer was demonstrated to be rescued by metabolic drugs, as metformin, a respiratory complex I blocker.

Combined, these data suggest a novel role for TRAP1 and oxidative metabolism in cancer progression and candidate targeting of mitochondrial bioenergetics as a strategy to improve cisplatin activity in human ovarian cancer [54].

## 6. Conclusions and Future Perspectives

The purpose of this review article was to present the dualistic role of TRAP1, as both an oncogene and oncosuppressor in human tumors, driven by different histotype-specific energetic metabolic behaviors (Figure 1). In support of this hypothesis, the switch from oxidative phosphorylation to glycolysis has been shown to be a very early event in hepatocarcinogenesis in a rat model, requiring the concomitant increase in the mitochondrial chaperone TRAP1 expression and the NRF2 transcription factor [25], associated with a decrease in SDH activity. This metabolic reprogramming was maintained in advanced hepatocellular carcinomas, along with high TRAP1 expression and low SDH activity in cancerous tissues, compared with the surrounding tissue. In this context, comparison of cancerous versus non-tumorigenic rat hepatocytes confirmed that the former had a much lower OCR and a significantly increased glycolytic profile [25]. A parallel yet opposite condition was observed in ovarian cancer, where TRAP1 expression decreased during tumor progression, whereas metabolism shifted toward a more oxidative context [26]. However, metabolic adaptation could also occur within the same tumor as a response to environmental stimuli, or as an evolutionary process. Therefore, it is possible that dynamic expression or activity regulation of TRAP1 and the signaling network that it helps to coordinate, reflects fluctuations in the energetic metabolic demands of tumor cells, with resultant changes in proliferation, spreading, nutrient uptake and response to stress and anticancer drugs. Metabolic remodeling is a general survival strategy suitable for either adaptation to hostile environments or resistance to anticancer agents. This leads to a link between chemoresistance and metabolic addiction that itself represents a promising drug target. Hence, targeting cancer metabolic networks, rather than single biomolecules, is emerging as a novel strategy for effective therapeutic approaches, alone or in combination therapies. These new vulnerabilities could be the key to eradicating the cancer stem cell population that are mainly responsible for relapse from first-line therapies. Moreover, the TRAP1/BRAF correlation suggests that targeting mitochondrial bioenergetics via the specific interference of these proteins may contribute to bypassing resistance mechanisms that allow tumors to escape the inhibition of canonical growth factor signaling.

## Figures and Tables

**Table 1:** Tumor Necrosis Factor Receptor-associated Protein 1 (TRAP1) is either positively or negatively selected in different tumors. Depending on the tumor type, TRAP1 expression is increased in the tumor compared to the normal tissues and further increases during progression and in metastatic sites or is lost in the later stages of the disease.

Tumor Type	TRAP1 Expression	References
Bladder Cancer	Inversely correlated with tumor stage	Yoshida et al. [16]
Breast Cancer	High in tumor cells compared to surrounding normal tissue	Zhang et al. [29] Maddalena et al. [32]
Clear Cell Renal Cell Carcinoma	Inversely correlated with tumor stage	Yoshida et al. [16]
Cervical Cancer	Inversely correlated with tumor stage	Yoshida et al. [16]
Colorectal Carcinoma	High in tumor cells compared to surrounding healthy mucosa, increased in advanced pathological stage, positively correlated with worse survival, upregulated at the transition between low- and high-grade adenomas	Costantino et al. [33], Pak et al. [34] Maddalena et al. [35]
Glioblastoma	High in tumor cells compared to adjacent normal astrocytes	Siegelin et al. [36]
Lung Cancer	High in tumor cells compared to adjacent normal bronchial mucosa	Im et al. [37]
Ovarian Cancer	Inversely correlated with stage and grade, positively correlated with better survival, reduced in peritoneal deposit distant from primary site	Aust et al. [24] Matassa et al. [26] Walker et al. [38] Amoroso et al. [39]
Prostate Cancer	High in both localized and metastatic prostate cancers compared to normal prostate or benign prostatic hyperplasia	Leav et al. [40] Lisanti et al. [41]
